# Overweight and obesity among women: analysis of demographic and health survey data from 32 Sub-Saharan African Countries

**DOI:** 10.1186/s12889-016-2698-5

**Published:** 2016-01-13

**Authors:** Subas Neupane, Prakash K.C., David Teye Doku

**Affiliations:** 1 School of Health Sciences, FI – 33014 University of Tampere, Tampere, Finland; 2 Department of Population and Health, University of Cape Coast, Private Mail Bag, University Post Office, Cape Coast, Ghana

**Keywords:** Overweight, Obesity, Women, BMI, DHS, Sub-Saharan Africa

## Abstract

**Background:**

Overweight and obesity are risk factors for many chronic diseases globally. However, the extent of the problem in low-income countries like Sub-Saharan Africa is unclear. We assessed the magnitude and disparity of both phenomena by place of residence, level of education and wealth quintile using cross-sectional data from 32 countries.

**Methods:**

Demographic and Health Survey (DHS) data collected in 32 Sub-Saharan African countries between January 2005 and December 2013 were used. A total of 250651 women (aged 15–49 years) were analyzed. Trained personnel using a standardized procedure measured body weight and height. Body mass index (BMI) was calculated by dividing body weight by height squared. Prevalence of overweight (25.0–29.9 kg/m^2^) and obesity (≥30.0 kg/m2) were estimated for each country. Analysis of the relationships of overweight and obesity with place of residence, education and wealth index were carried out using logistic regression.

**Results:**

The pooled prevalence of overweight for the region was 15.9 % (95 % CI, 15.7–16.0) with the lowest in Madagascar 5.6 % (95 % CI, 5.1–6.1) and the highest in Swaziland 27.7 % (95 % CI, 26.4–29.0). Similarly, the prevalence of obesity was also lowest in Madagascar 1.1 % (95 % CI, 0.9–1.4) and highest in Swaziland 23.0 (95 % CI, 21.8–24.2). The women in urban residence and those who were classified as rich, with respect to the quintile of the wealth index, had higher likelihood of overweight and obesity. In the pooled results, high education was significantly associated with overweight and obesity.

**Conclusions:**

The prevalence of overweight and obesity varied highly between the countries and wealth index (rich vs. poor) was found to be the strongest predictor in most of the countries. Interventions that will address the socio-cultural barriers to maintaining healthy body size can contribute to curbing the overweight and obesity epidemic in Africa.

## Background

Obesity has become a major public health problem both in developed and developing countries [1]. Overweight and obesity are the fifth leading risk for global deaths [2]. At least 2.8 million adults die each year as a result of being overweight or obese [3]. In addition, 44 % of the diabetes burden, 23 % of the ischemic heart disease burden and between 7 and 41 % of certain cancer burdens are attributable to overweight and obesity [4]. The World Health Organization (WHO) defines a person to be overweight if his or her body mass index (BMI) is >25, and obese if BMI is ≥30 [5].

According to a recent research reports, the burden of obesity is increasing in developing countries and also no significant reduction has been seen in its burden in developed countries over the past few decades [1]. For example, in West Africa, the prevalence of obesity rapidly increased during the last two decades of the 20th century and it continues to increase in the 21st century. A recent review indicates that the prevalence of obesity in the region between 2000 and 2004 was roughly 10 % [6, 7].

Overweight and obesity is as a result of energy imbalance that consumes more calories than what is equivalently expended in physical activities. Westernization and Urbanization are said to be among the main reasons for this energy imbalance in the African region and they are viewed from two perspectives [7]. First, urbanization and westernization lead to decreased physical activity. Second, urbanization and westernization result in increased food supply, which include access to high caloric fast foods and sugar sweetened beverages (fatty foods) [8]. Previous studies have found socioeconomic differences in obesity and overweight [9], and in developing countries the direction is not consistent [9]. While some studies found the likelihood of being obese or overweight to the detriment of those with higher socio-economic status (SES) [10, 11], others reported higher chances of being overweight or obese among those with lower SES [12]. Height, which is a component of BMI is itself socioeconomically patterned because past socioeconomic status and nutrition during the developmental stages of life (childhood and adolescence) affect height.

Over all, some few studies on obesity in Africa have been conducted [13–15]. While these studies have made various contributions to understanding the phenomenon in the region, their focuses differ from the present studies. For instance, Steyn and Mchiza [13] focused on understanding whether changes in nutrition diet and obesity have taken place over the past three decades in Sub-Saharan Africa. Ziraba et al. [14] on the other hand, explored the phenomenon in urban Africa. Another challenge with these previous findings is that they used different data sets, which have varying representativeness and methodological procedures. Therefore, the findings are not comparable across countries. To address these constraints, this study used nationally representative data from 32 Sub-Saharan African countries with similar method of data collection to explore the prevalence of overweight and obesity in the region. Furthermore, the study explored disparity in the phenomenon by place of residence, level of education and wealth quintile.

## Methods

### Data sources and procedures

Thirty-two nationally representative cross-sectional data from the most recent Demographic and Health survey (DHS) conducted between January 1, 2005, and December 31, 2013 in Sub-Saharan Africa were used. The DHS survey data were collected at about 5-year intervals across low and middle-income countries. DHS collect data on health and welfare by interviewing women of reproductive age (15–49 years), their children, and their households. In this analysis only women who had information on age and height were included. DHS surveys are available to investigators through the World Wide Web (http://www.dhsprogram.com). All 32 countries, DHS survey followed the same standard procedures. Detail descriptions of DHS sampling procedures, validation of questionnaire, and data collection methods are published elsewhere (http://www.dhsprogram.com). Briefly, the DHS used a stratified two-stage random sampling approach, consisting of a selection of census enumeration areas based on a probability, followed by a random selection of household from a complete listing of a household within the selected enumeration areas. In this study, all together 366885 women from 32 countries responded to the surveys with the response rates varying from 86.2 to 100.0 %. However, the present analysis is based on all women who had information on weight and height (*N* = 250651). Women granted written informed consent before interviewing them. Ethical approval was given by ICF International (Calverton, MD, USA) institutional review board and by individual review boards within every participating country.

### Measurements of variables

In all DHS survey, trained personnel measured the height and weight using a standardized procedure. Weight was measured using solar-powered scales with accuracy of 0.1 kg and height was measured using standardized measuring boards with accuracy to 0.1 cm. Body mass index (BMI) was calculated by dividing body weight (kg) by squared height (m^2^). Overweight and obesity were defined as recommended by World Health Organization [5]: overweight 25.0–29.9 kg/m^2^ and obesity ≥30.0 kg/m^2^. As the prevalence of obesity was low (<1 %) in some countries, the categories of overweight and obesity were combined together in the logistic regression analyses. Only the respondents who had information on BMI were included in the analyses.

The participants’ place of residence was designated as rural and urban according to country specific definitions. The wealth index was calculated using easy-to-collect data on a household’s ownership of selected assets (e.g. televisions, bicycles, cars, materials used for housing construction and types of water access and sanitation facilities). The wealth index was then generated as a composite variable by demographic and health survey (DHS) staff using principal components analysis. Continuous scale of relative wealth was then categorized into five (poorest, poorer, middle, richer, and richest) according to the quintile of the sample. Wealth index was not available from the DHS data from Chad. Maternal education was assessed from self-report of the completed educational level (no education, primary, secondary, or higher).

### Statistics

Sample weights were applied to the data to remove the bias due to unequal selection probabilities. Descriptive figures of the study participants are reported in percentages and the prevalence of overweight and obesity with their 95 % confidence intervals (CIs) are reported separately for each country. The pooled prevalence for the region was also estimated. Scatterplots were used to visualize the relationship between no education and overweight and obesity with the size of a marker relative to the number of outcome events to reflect their influence on the correlation. Analyses of the relationships between overweight and obesity and place of residence, education and wealth index were carried out. Odds ratios (ORs) and their 95 % confidence intervals (CIs) for overweight and obesity (<25.0 = 0, ≥ 25.0 = 1) were estimated in multivariate logistic regressions model including the place of residence (urban = 0, rural =1), maternal education (secondary or higher =0, no or primary education =1) and wealth index (richer or richest =0, poorest to middle =1). All the analyses were performed using the SPSS statistical software package version 21 and Stata version 13.

## Results

Data from a total of 250651 women from 32 countries in Sub-Saharan Africa were analysed in this study. Table 1 shows the country level characteristics of the study participants. The mean age of the studied women varied from 27.68 years (Comoros) to 29.43 years (Liberia). The overall mean age of the study participants was 28.46 years. Only 10.5 % of women were urban residents in Burundi which is the least among countries studied whereas Congo (Brazzaville) had the highest number of urban residents (66.8 %). On the average, 36.6 % of the women resided in urban areas. According to wealth index, Lesotho had the fewest poorest (14.6 %) and the highest number of poorest women were found in Burundi (20 %). Similarly, Burundi also had the fewer richest (20 %) people compared to Kenya (26.2 %). The largest number of women who had no education (80.7%) were found in Niger compared to only 1.2 % in Lesotho. Similarly, women in Lesotho also had the lowest mean number of children (1.82) compared to 4.42 % in Chad. The pooled prevalence of overweight for the region was 15.9 % (95 % CI, 15.7–16.0) with the lowest prevalence in Madagascar 5.6 % (95 % CI, 5.1–6.1) and the highest in Swaziland 27.7 % (95 % CI, 26.4–29.0) Table 2. Other countries such as Burkina Faso, Burundi, Chad and Ethiopia had also lower prevalence (<10 %) of overweight. Moreover, Cameroon, Comoros, Gabon, Sao Tome, and Zimbabwe were among those with high prevalence (>20 %) of overweight among women. Similarly, the lowest and the highest prevalence of obesity were also found in the same countries; lowest in Madagascar 1.1 % (95 % CI, 0.9–1.4) and the highest in Swaziland 23.0 (95 % CI, 21.8–24.2). Many countries such as Burkina Faso, Burundi, Chad, Congo Republic, Guinean, Malawi, Niger, Rwanda, Tanzania and Zambia have obesity prevalence of less than 5 %.

**Table 1:** Characteristics of women in Demographic Health Surveys across countries

Country	N^a^	Mean age (years)	Urban residents (%)	Lowest house-hold wealth quintile (%)	Highest house-hold wealth quintile (%)	No education (%)	Mean value of total children ever born
Benin, 2006	16071	28.93	45.6	17.1	23.8	60.0	2.77
Burkina Faso, 2010	8503	28.72	26.7	18.2	24.4	74.0	3.27
Burundi, 2010	4616	27.74	10.5	20.0	20.0	45.4	2.74
Cameroon, 2011	7872	27.82	54.0	15.8	24.3	19.0	2.73
Chad	3801	28.26	18.5	NA	NA	77.1	4.42
Comoros	5155	27.68	33.4	16.3	22.0	31.2	2.19
Congo (Brazzaville) 2011-12	5480	28.48	66.8	17.7	20.3	5.7	2.54
Congo Republic, 2007	4763	28.52	45.0	18.7	21.9	22.1	3.08
Cote d’Ivoire, 2011-12	4694	28.20	49.6	18.0	23.6	54.4	2.70
Ethiopia, 2011	16065	27.70	23.3	18.2	25.1	51.0	2.91
Gabon, 2012	5408	28.68	88.4	14.8	21.9	4.5	2.35
Ghana, 2008	4822	28.99	48.5	15.8	23.1	21.2	2.33
Guinean, 2005	4756	28.16	35.0	17.5	22.5	66.4	2.95
Kenya, 2008	8295	28.44	25.3	16.5	26.2	8.8	2.68
Lesotho, 2009	3903	28.19	32.6	14.6	26.0	1.2	1.82
Liberia, 2007	6951	29.43	42.1	17.7	22.0	42.7	3.11
Madagascar, 2008-09	8389	28.84	16.9	17.9	24.2	19.2	2.88
Malawi, 2010	7554	27.95	19.6	16.9	24.4	15.2	3.06
Mali, 2006	5261	28.77	24.2	19.9	22.9	75.0	3.35
Mozambique, 2011	13619	28.58	34.5	19.0	23.4	31.3	2.90
Namibia, 2006-07	9446	28.31	47.7	16.9	22.9	6.7	1.92
Niger, 2006	5149	28.83	17.6	17.7	21.3	80.7	4.22
Nigeria, 2013	38263	28.81	42.1	18.2	22.8	37.7	3.07
Rwanda, 2010	6948	28.23	15.2	18.1	21.9	15.3	2.38
Sao Tome, 2008-09	2246	29.02	63.3	17.5	24.1	5.9	2.73
Senegal, 2010-11	5768	27.97	49.1	16.3	24.1	57.4	2.73
Sierra Leon, 2008	3487	29.26	34.7	18.4	21.4	67.8	3.08
Swaziland, 2006-07	4853	27.73	26.1	15.8	24.9	8.0	2.28
Tanzania, 2010	10002	28.58	28.4	16.7	23.6	18.9	2.89
Uganda, 2011	2666	27.94	20.6	17.3	25.7	12.2	3.43
Zambia, 2007	7037	28.08	42.1	17.5	25.0	10.4	3.04
Zimbabwe, 2010-11	8806	28.17	37.7	17.1	24.4	2.3	2.11

^a^ Number of women of reproductive age (15–49 years) group who had information on BMI

*NA* data not available

**Table 2:** Prevalence of overweight and obesity with their 95 % confidence intervals (CIs) among women by countries

Country	Prevalence of overweight (95 % CI)	Prevalence of obesity (95 % CI)
Benin, 2006	19.4 (18.8–20.0)	6.4 (6.0–6.8)
Burkina Faso, 2010	8.0 (7.5–8.6)	2.8 (2.4–3.1)
Burundi, 2010	8.3 (7.5–9.1)	2.3 (1.9–2.8)
Cameroon, 2011	22.0 (21.0–22.9)	10.2 (9.5–10.9)
Chad	8.9 (8.0–9.8)	2.5 (2.0–3.0)
Comoros	25.3 (24.1–26.5)	13.1 (12.2–14.1)
Congo (Brazzaville) 2011–12	14.6 (13.7–15.5)	5.7 (5.1–6.4)
Congo Republic, 2007	10.4 (9.6–11.3)	2.6 (2.1–3.0)
Cote d’Ivoire, 2011–12	17.6 (16.5–18.7)	5.9 (5.3–6.6)
Ethiopia, 2011	6.2 (5.8–6.6)	1.7 (1.5–2.0)
Gabon, 2012	23.6 (22.5–24.7)	14.9 (13.9–15.8)
Ghana, 2008	19.8 (18.6–20.9)	8.5 (7.7–9.3)
Guinean, 2005	14.5 (13.5–15.6)	4.4 (3.8–5.0)
Kenya, 2008	17.9 (17.0–18.7)	7.5 (6.9–8.0)
Lesotho, 2009	24.5 (23.1–25.8)	15.8 (14.7–16.9)
Liberia, 2007	15.4 (14.6–16.3)	5.6 (5.1–6.2)
Madagascar, 2008–09	5.6 (5.1–6.1)	1.1 (0.9-1.4)
Malawi, 2010	13.5 (12.7–14.3)	3.2 (2.8–3.6)
Mali, 2006	13.5 (12.6–14.4)	5.8 (5.2–6.5)
Mozambique, 2011	15.1 (14.5–15.7)	5.3 (4.9–5.6)
Namibia, 2006–07	16.3 (15.6–17.0)	11.6 (11.0–12.3)
Niger, 2006	15.2 (14.3–16.2)	4.9 (4.3–5.5)
Nigeria, 2013	17.8 (17.3–18.1)	7.6 (7.3–7.8)
Rwanda, 2010	15.6 (14.7–16.4)	2.4 (2.0–2.7)
Sao Tome, 2008–09	23.0 (21.3–24.7)	11.2 (10.0–12.5)
Senegal, 2010–11	13.3 (12.4–14.2)	5.1 (4.5–5.7)
Sierra Leon, 2008	21.3 (20.0–22.7)	9.9 (8.9–10.9)
Swaziland, 2006–07	27.7 (26.4–29.0)	23.0 (21.8–24.2)
Tanzania, 2010	15.5 (14.8–16.2)	6.2 (5.7–6.7)
Uganda, 2011	15.3 (14.0–16.7)	4.6 (3.8–5.4)
Zambia, 2007	15.0 (14.1–15.8)	4.6 (4.1–5.1)
Zimbabwe, 2010–11	20.7 (19.9–21.6)	9.9 (9.3–10.6)
Pooled prevalence for the region	15.9 (15.7–16.0)	6.7 (6.6–6.8)

**Fig. 1:**
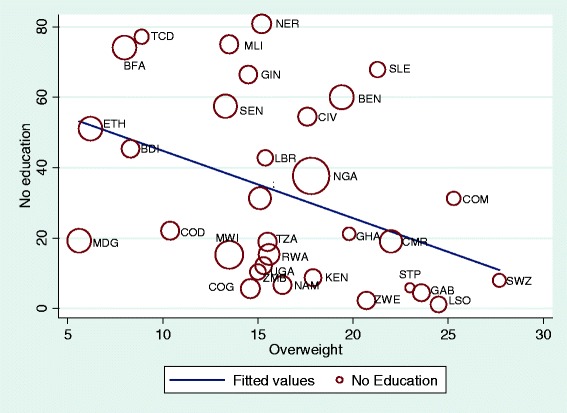
Scatterplot of overweight prevalence of women in each country by no education (weighted by total sample size of the country). *Abbreviation*: *BEN* Benin, *BFA* Burkina Faso, *BDI* Burundi, *CMR* Cameron, *TCD* Chad, *COM* Comoros, *COG* Congo (Brazzaville), *COD* Congo Republic, *CIV* Cote d’Ivorie, *ETH* Ethiopia, *GAB* Gabon, *GHA* Ghana, *GIN* Guinean, *KEN* Kenya, *LSO* Lesotho, *LBR* Liberia, *MDG* Madagascar, *MWI* Malawi, *MLI* Mali, *MOZ* Mozambique, *NAM* Namibia, *NER* Niger, *NGA* Nigeria, *RWA* Rwanda, *STP* Sao Tome, *SEN* Senegal, *SLE* Sierra Leon, *SWZ* Swaziland, *TZA* Tanzania, *UGA* Uganda, *ZMB* Zambia, *ZWE* Zimbabwe

**Fig. 2:**
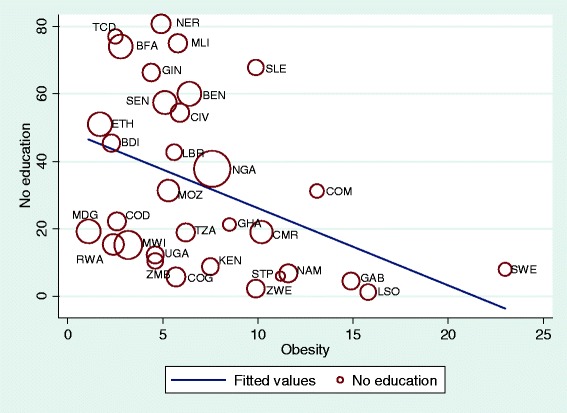
Scatterplot of obesity prevalence of women in each country by no education (weighted by total sample size of the country). *Abbreviation*: *BEN* Benin, *BFA* Burkina Faso, *BDI* Burundi, *CMR* Cameron, *TCD* Chad, *COM* Comoros, *COG* Congo (Brazzaville), *COD* Congo Republic, *CIV* Cote d’Ivorie, *ETH* Ethiopia, *GAB* Gabon, *GHA* Ghana, *GIN* Guinean, *KEN* Kenya, *LSO* Lesotho, *LBR* Liberia, *MDG* Madagascar, *MWI* Malawi, *MLI* Mali, *MOZ* Mozambique, *NAM* Namibia, *NER* Niger, *NGA* Nigeria, *RWA* Rwanda, *STP* Sao Tome, *SEN* Senegal, *SLE* Sierra Leon, *SWZ* Swaziland, *TZA* Tanzania, *UGA* Uganda, *ZMB* Zambia, *ZWE* Zimbabwe

**Fig. 3:**
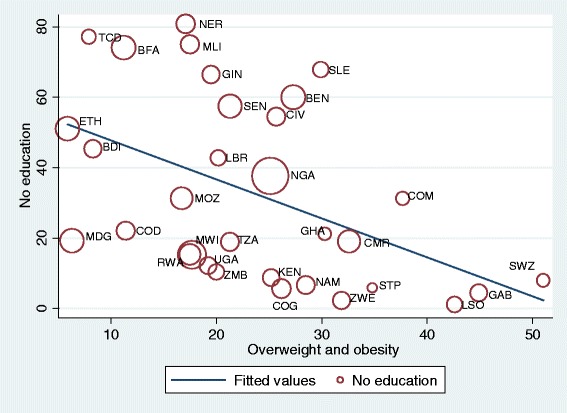
Scatterplot of overweight obesity prevalence of women in each country by no education (weighted by total sample size of the country). *Abbreviation*: *BEN* Benin, *BFA* Burkina Faso, *BDI* Burundi, *CMR* Cameron, *TCD* Chad, *COM* Comoros, *COG* Congo (Brazzaville), *COD* Congo Republic, *CIV* Cote d’Ivorie, *ETH* Ethiopia, *GAB* Gabon, *GHA* Ghana, *GIN* Guinean, *KEN* Kenya, *LSO* = Lesotho, *LBR* = Liberia, *MDG* Madagascar, *MWI* Malawi, *MLI* Mali, *MOZ* Mozambique, *NAM* Namibia, *NER* Niger, *NGA* Nigeria, *RWA* Rwanda, *STP* Sao Tome, *SEN* Senegal, *SLE* Sierra Leon, *SWZ* Swaziland, *TZA* Tanzania, *UGA* Uganda, *ZMB* Zambia, *ZWE* Zimbabwe

Figures 1, 2 and 3 show clear substantial heterogeneity in prevalence estimates of overweight and obesity between countries of Sub-Saharan Africa.

Table 3 presents the odds ratio and 95 % CI for overweight and obesity among women in urban vs. rural, high education vs. low and  vs. poor . The place of residence, education and wealth index strongly predicted overweight and obesity among Sub-Saharan African women. Significantly, the largest variation of overweight and obesity was between urban and rural (OR 7.94, 95 % CI, 6.95–9.07), high vs. low education (OR 3.71, 95 % CI, 3.27–4.21) and rich vs. poor (OR 7.86, 95 % CI, 6.66–9.28) in Ethiopia. Likewise, the least variation of overweight and obesity was by urban vs. rural (OR 1.24, 95 % CI, 1.10–1.40) in Gabon, high vs. low education (OR 0.65, 95 % CI, 0.58–0.73) and rich vs. poor (OR 1.31, 95 % CI, 1.17–1.47) in Comoros. Urban residents compared to rural had higher odds of overweight and obesity. Also, in many countries women with high education had higher odds of overweight and obesity except in Benin, Comoros, Sao Tome and Senegal. In almost all studied countries the overweight and obesity was more common among rich women compared to poor with an average odds of 2.45 (95 % CI 2.40–2.50).

**Table 3:** Odds ratios and their 95 % confidence intervals (CIs) for overweight and obesity among women due to place of residence, education and wealth index across countries

Country	Percentages^a^	OR (95 % CI)		
		Residence, urban vs. rural	Education, high vs. low	Wealth index, rich vs. poor
Benin, 2006	27.3	1.66 (1.55–1.79)	0.96 (0.88–1.05)	2.10 (1.95–2.26)
Burkina Faso, 2010	11.2	4.27 (3.70–4.92)	2.55 (2.16–3.02)	4.46 (3.80–5.22)
Burundi, 2010	8.3	5.75 (4.72–6.99)	3.11 (2.54–3.82)	4.01 (3.24–4.97)
Cameroon, 2011	32.6	2.25 (2.04–2.48)	1.66 (1.51–1.82)	2.51 (2.28–2.76)
Chad	7.9	5.16 (4.05–6.57)	3.30 (2.48–4.40)	NA
Comoros	37.7	1.44 (1.29–1.63)	0.65 (0.58–0.73)	1.31 (1.17–1.47)
Congo (Brazzaville) 2011–12	26.2	2.55 (2.24–2.92)	1.91 (1.66–2.19)	3.21 (2.78–3.69)
Congo Republic, 2007	11.4	2.56 (2.14–3.06)	1.92 (1.62–2.28)	2.74 (2.30–3.28)
Cote d’Ivoire, 2011–12	25.7	2.56 (2.14–3.06)	1.14 (0.96–1.35)	2.65 (2.31–3.04)
Ethiopia, 2011	5.9	7.94 (6.95–9.07)	3.71 (3.27–4.21)	7.86 (6.66–9.28)
Gabon, 2012	44.9	1.24 (1.10–1.40)	1.06 (0.95–1.18)	1.73 (1.53–1.96)
Ghana, 2008	30.3	3.01 (2.64–3.43)	1.75 (1.54–1.99)	4.12 (3.61–4.70)
Guinean, 2005	19.5	3.02 (2.60–3.50)	1.53 (1.29–1.83)	3.30 (2.82–3.85)
Kenya, 2008	25.2	2.49 (2.25–2.76)	2.07 (1.87–2.29)	3.13 (2.82–3.48)
Lesotho, 2009	42.6	1.65 (1.43–1.91)	1.32 (1.16–1.50)	2.07 (1.82–2.36)
Liberia, 2007	20.2	1.95 (1.73–2.19)	1.61 (1.41–1.83)	2.22 (1.98–2.50)
Madagascar, 2008–09	6.3	3.28 (2.76–3.90)	2.91 (2.44–3.46)	4.66 (3.80–5.71)
Malawi, 2010	17.7	2.14 (1.84–2.50)	1.61 (1.40–1.86)	2.19 (1.94–2.48)
Mali, 2006	17.5	3.34 (2.90–3.84)	1.44 (1.21–1.72)	3.10 (2.68–3.59)
Mozambique, 2011	16.7	3.02 (2.77–3.30)	1.84 (1.67–2.01)	4.53 (4.10–5.01)
Namibia, 2006–07	28.5	2.14 (1.95–2.34)	1.27 (1.15–1.40)	2.76 (2.52–3.03)
Niger, 2006	17.1	3.97 (3.44–4.57)	1.77 (1.46–2.15)	3.18 (2.74–3.69)
Nigeria, 2013	25.1	2.11 (2.02–2.21)	1.67 (1.59–1.75)	2.90 (2.76–3.04)
Rwanda, 2010	17.5	2.10 (1.82–2.43)	1.68 (1.45–1.95)	2.33 (2.05–2.64)
Sao Tome, 2008–09	34.8	1.29 (1.09–1.53)	0.79 (0.66–0.96)	1.43 (1.20–1.70)
Senegal, 2010–11	21.3	2.18 (1.90–2.50)	0.77 (0.65–0.93)	1.90 (1.66–2.19)
Sierra Leon, 2008	29.9	1.59 (1.38–1.84)	1.29 (1.10–1.53)	1.38 (1.19–1.59)
Swaziland, 2006–07	51.0	1.32 (1.16–1.49)	1.13 (1.01–1.27)	1.63 (1.46–1.83)
Tanzania, 2010	21.3	2.64 (2.38–2.94)	1.56 (1.41–1.74)	3.62 (3.27–4.01)
Uganda, 2011	19.2	3.20 (2.63–3.89)	2.18 (1.79–2.65)	4.68 (3.77–5.82)
Zambia, 2007	20.0	2.72 (2.41–3.07)	1.78 (1.58–2.01)	3.15 (2.78–3.57)
Zimbabwe, 2010–11	31.9	2.06 (1.88–2.26)	1.16 (1.05–1.28)	2.18 (1.98–2.39)
Pooled value for the region	22.5	2.35 (2.31–2.40)	1.81 (1.78–1.85)	2.45 (2.40–2.50)

*NA* data not available

^a^Percentages of overweight and obesity cases

## Discussion

A number of previous studies have reported the emergence of obesity and overweight in developing countries [1, 10, 16, 17]. Although these earlier studies provide aggregate picture of the epidemic in developing countries, they lack in-depth analysis of the phenomenon in specific regions, alongside socio-demographic dimensions. Furthermore, with the exception of few studies [1, 10], the previous studies used data collected from different sources using varied methodologies and therefore it was difficult for comparison across the diverse regions within the developing countries. An additional constraint of a number of the previous studies is that the BMI was estimated from self-reported weight and height and therefore it may be biased by the social desirability regarding weight in the respective countries and communities within these countries. To build on these previous studies and to address the aforementioned gaps, the present study used a large sample of the data which were collected using similar methods among women aged 15 to 49 years in 32 African countries to explore the prevalence of overweight and obesity in the region and to investigate the socio-demographic disparities in the two outcomes across the region.

Our study found high variation in overweight and obesity problem across Sub-Saharan African countries. Twenty-seven of the studied countries had overweight prevalence higher than 10 % and 7 countries had more than 10 % prevalence of obesity. Urban residents, women with high education and rich women as measured by wealth index quintile had higher odds of overweight and obesity in general. These findings put these countries at the risk of high burden of obesity related morbidity and mortality in the future.

There exist some systematic reviews and meta-analyses [1, 6, 9, 12] therefore we did not perform a systematic review of the scientific literature. However we search the recent literature published in English, employing no date restrictions. Search terms included BMI, overweight, obesity, developing countries, Sub-Saharan Africa etc. We identified 20 relevant primary research articles based on low- and middle-income countries. Only very few of them were based on national data.

Considerable differences in overweight and obesity across Sub-Saharan Africa were found. This ranged from low overweight and obesity of 6.7 % in Madagascar to 50.8 % in Swaziland. Unlike in previous studies [10, 18] where the characteristics of overweight and obesity have been associated with the level of development in the countries, it seems that the prevalence in the present study does not reflect the developmental level of countries. The emerging prevalence of overweight and obesity in Africa has been largely attributed to the rising level of urbanisation in the region and its attendant global nutrition transition [19]. Urbanization in Africa is increasing rapidly, for example, for the first time, the Ghana Population and Housing Census shows that more Ghanaians are living in urban areas than rural areas [20]. African countries are projected to have 50 % urbanisation by 2020 [21]. The growing urbanization comes along with sedentary lifestyle, "motorised culture", availability of refined foods and physical inactivity related recreations such as cinema houses and video games are all implicated in the rising overweight and obesity in Sub-Saharan Africa. These risk factors for obesity are similar to those found in several studies across the world as accounting for the increasing overweight and obesity epidemic in developing countries and the stall in the phenomenon in developed countries [22–25].

Our study found differences in overweight and obesity to the disadvantage of those in urban settings similar to a recent study [26]. These rural-urban disparities could be explained by the differences in lifestyle and dietary pattern between urban and rural dwellers in Africa. In rural areas, residents mainly eat fresh food from the farm, and mostly green and fresh fruits and vegetables are available from backyards. Therefore, the dietary pattern of rural folks, although unintentional, tends to be healthy compared to that of urban folks. Apart from diet, the lifestyle of urban residents is tilted towards westernization and the blind adoption of the so-called western lifestyle. There are growing numbers of western food outlets and urban residents perceive eating at such joints as a sign of affluence. Transnational fast-food companies in the region are aggressively exploiting this perception [21].

The nature of occupation prevailing in rural and urban setting in Africa also contributes to the differences in obesity and overweight between the two settings. In most rural settings the main form of occupation is still non-mechanized agriculture and physical activity based vocations such as fishing, small scale mining and lumbering. An occupation related physical activity is a known protective factor against obesity [27]. In the urban area, however, there is increasing shift from high physical activity based occupations such as construction and working in factories to sedentary and service based occupation. On the other hand, the growing urbanisation coupled with climate change and other socioeconomic transition have raised food prices in most developing countries and it is possible that rural women are just unable to afford enough food to feed on [28, 29].

We also found socioeconomic differences in overweight and obesity by level of education and wealth to the detriment of those with lower socioeconomic status in most countries across Africa similar to those found in other previous studies [12, 30]. Studies have argued that cultural norms that favour fatter body size contribute significantly to the SES differences in overweight and obesity in developing countries, particularly in Africa [31]. Women of higher SES may have the resources and knowledge of the importance of physical activity and healthy diet but they also face several socio-cultural barriers that may prevent them from putting those into use [10, 30].

Reporting national-level rates of overweight and obesity using nationally representative data is a major strength of our study, therefore the findings are generalizable to the respective countries. In DHS surveys, each country followed standardised procedure to collect the data using validated questionnaire, therefore the data are comparable between the countries. The data on the body weight and height was measured by trained study personnel with similar measurement equipment which is another strengths of our study. There are also some limitations in this study which are worth discussing. Firstly, we did not have data on waist circumference which would have allowed examination of trends in abdominal obesity. Additionally, no data were available on behavioural or other factors that could have explained the prevalence of overweight across the countries.

## Conclusions

The prevalence of overweight and obesity varied highly between the countries and wealth index (rich vs. poor) was found to be the strongest predictor in most of the countries. Owing to the well-known risk of overweight and obesity, the emerging epidemic in Africa needs to be addressed early enough to prevent its related morbidity and mortality. A number of interventions can be implemented. The fast growing cities should include in their development plans the creation of safe pedestrian walkways and bicycle lanes to enable pedestrians and cyclist feel safe. This may encourage walk and cycling in cities. There should be national and regional campaigns to encourage consumptions of healthy foods and physical activity. This should include the provision of healthy foods at school and nutritional education in schools and communities. Interventions that will aim at addressing the socio-cultural barriers to maintaining healthy body size can also contribute to fighting the overweight and obesity epidemic in Africa.
